# In Situ Synthesis of MnMgFe-LDH on Biochar for Electrochemical Detection and Removal of Cd^2+^ in Aqueous Solution

**DOI:** 10.3390/molecules27227875

**Published:** 2022-11-15

**Authors:** Yongfang Yu, Wenting Yang, Haocheng Wang, Guoqin Huang

**Affiliations:** Key Laboratory of Crop Physiology, Ecology and Genetic Breeding, Ministry of Education, Research Center on Ecological Sciences, Jiangxi Agricultural University, Nanchang 330045, China

**Keywords:** layered double hydroxides, biochar, cadmium, electrochemical determination, adsorption

## Abstract

Herein, MnMgFe-layered double hydroxides/biochar (MnMgFe-LDHs/BC) composite was fabricated by immobilizing MnMgFe-LDHs on BC via the coprecipitation method, which was employed as an effective material for the detection and removal of Cd^2+^ from aqueous media. A lamellar structure of MnMgFe-LDHs with abundant surface-hydroxyl groups and various interlayer anions inside present a greater chance of trapping Cd^2+^. Meanwhile, the conductive BC with a porous structure provides numerous channels for the adsorption of Cd^2+^. Using the MnMgFe-LDHs/BC-based sensor, Cd^2+^ can be detected with a low limit of detection down to 0.03 ng/L. The feasibility of detecting Cd^2+^ in paddy water was also carried out, with satisfactory recoveries ranging from 97.3 to 102.3%. In addition, the MnMgFe-LDHs/BC material as an adsorbent was applied to remove Cd^2+^ from water with adsorption capacity of 118 mg/g, and the removal efficiency can reach 91%. These results suggest that the as-prepared MnMgFe-LDHs/BC can serve as a favorable platform for efficient determination and removal of Cd^2+^ in water.

## 1. Introduction

Nowadays, heavy metal ions (HMIs) pollution have drawn great attention due to their serious harm. As one of the most common HMIs, Cd^2+^ shows a great threat to the human body due to its high toxicity and non-biodegradability [1,2]. Long-term exposure to Cd^2+^ could cause various disorders, including damage to the liver, kidney, lung, and bone, and even cause cancers [3,4]. What’s more, Cd^2+^ in water and soil will enter into the human body through the food chain and can be accumulated. Therefore, the sensitive detection and efficient removal of Cd^2+^ are extremely urgent for human health and ecological balance.

Up to the present, many analytical techniques have been developed to detect Cd^2+^. Among these techniques, electrochemical methods stand out for a series of reliable advantages such as easy operation, economy, easy miniaturization, and rapid detection [5,6]. For electrochemical sensors, the performance directly depends on the properties of the constructed electrode interface. So, it is necessary to develop materials with a large specific surface and abundant metal ion adsorption sites in which HMIs can be enriched on the surface of an electrode and the sensitivity of the sensor can be improved. Biochar (BC), a carbon-rich adsorbent with interesting physical and chemical characteristics, is produced by pyrolysis of biomass in an inert atmosphere. Due to its eco-friendliness, pore structure, and high surface area, BC has great potential for electrochemical determination and removal of HMIs [7,8,9,10]. Nonetheless, the limited functional groups restrict its adsorption capacity. So, some modification and functionalization are necessary to endow BC with an outstanding adsorption performance.

Layered double hydroxides (LDHs) are a kind of anionic layered compound assembled from positively charged host layers and interlayer anions through noncovalent bond interactions. The general form of LDHs is presented as [M^2+^_(1−X)_ M^3+^_X_ (OH^−^)_2_]^X+^ (A^n−^)_x/n_∙mH_2_O, where M^2+^ and M^3+^ are divalent and trivalent metal ions, x is their molar ratio, A^n−^ is organic and inorganic interlayer anionic groups, and m refers to the molecule number of hydrate H_2_O [11,12]. These materials show strong adsorption capacity for cations in water through the surface-induced precipitation and substitution of metal ions. At present, the related research mainly focus on bimetallic LDHs [13,14,15]; few studies on the adsorption behaviors of ternary LDH have been performed. For example, MnMgFe-LDHs with hydrotalcite-like structures have been utilized for the adsorption of Pb^2+^ and Cd^2+^, demonstrating higher adsorption capacity than bimetallic LDHs [16,17]. Furthermore, in terms of electrochemistry, LDH materials have also been developed into various sensors. Zhang et al. reported the use of MgFe-LDH microsphere/graphene for simultaneous electrochemical determination of Pb^2+^ and Cd^2+^ [18]. Xu el al. used MgAl-LDH flower as the electrochemical sensing platform for nitrite detection [19]. However, there are few reports about applying the assembly of MnMgFe-LDHs and BC for simultaneous detection and removal of Cd^2+^.

In this study, MnMgFe-LDHs/BC composite has been synthesized with a facile coprecipitation method, which was employed for electrochemical detection and removal of Cd^2+^ from aqueous solution. The abundant surface-hydroxyl groups and interlayer anions of MnMgFe-LDHs would provide more functional sites for trapping Cd^2+^. Moreover, the hierarchical porosity, outstanding conductivity, and abundant oxygen-containing functional groups of BC would further improve the electron transfer rate and enrichment of Cd^2+^ on the electrode surface. Attributing to the synergistic effects between MnMgFe-LDHs and BC, the sensor based on MnMgFe-LDHs/BC displayed a low limit of detection of 0.03 ng/L toward Cd^2+^. Furthermore, satisfactory removal performance toward Cd^2+^ using MnMgFe-LDHs/BC as the adsorbent was achieved as well.

## 2. Results and Discussion

### 2.1. Morphology and Structure Characterization

**Figure 1:**
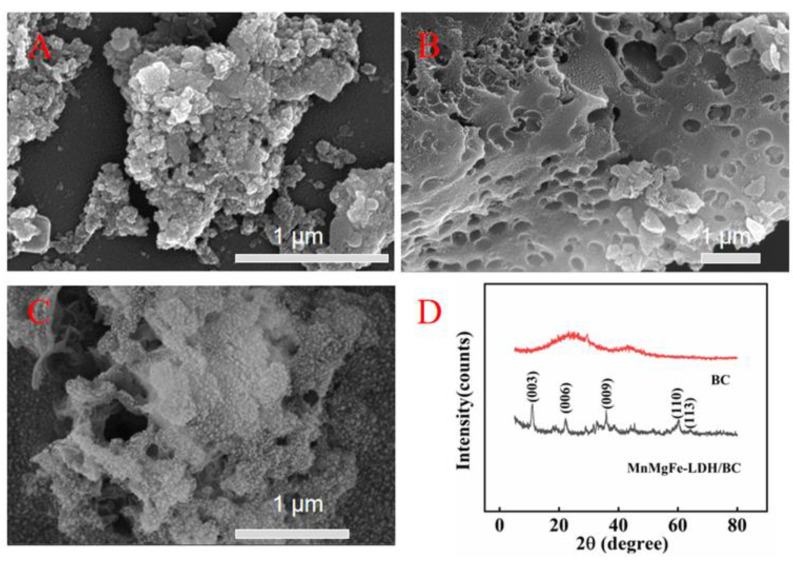
SEM of (**A**) MnMgFe-LDHs, (**B**) BC, and (**C**) MnMgFe-LDHs/BC; (**D**) XRD patterns of BC and MnMgFe-LDHs/BC.

The morphology and microstructure of MnMgFe-LDHs, BC, and MnMgFe-LDHs/BC were observed via SEM. As shown, MnMgFe-LDHs displays a blocky structure composed of MnMgFe-LDHs particles (Figure 1A). On the other hand, BC presents a well-defined porous structure with a rough surface (Figure 1B). For MnMgFe-LDHs/BC (Figure 1C), it is clearly seen that MnMgFe-LDHs particles are well-dispersed on the BC. The crystalline phases of BC and MnMgFe-LDHs/BC were characterized by XRD (Figure 1D). For BC, two diffraction peaks at about 21°and 27.3° are attributed to (002) and (111) diffraction planes of graphite lattice, respectively [20]. After the formation of MnMgFe-LDHs/BC composite, several new diffraction peaks were observed at 2θ = 11.6, 23.3, 33.6, 34.4, 39, 46.4, 59.75, and 61.2°, ascribing to the (003), (006), (009), (110), and (113) crystal phases of MnMgFe-LDHs, which is coincidental with that previously reported in [16]. Therefore, SEM and XRD analysis results indicate the successful combination of MnMgFe-LDHs and BC.

### 2.2. Electrochemical Characterizations

**Figure 2:**
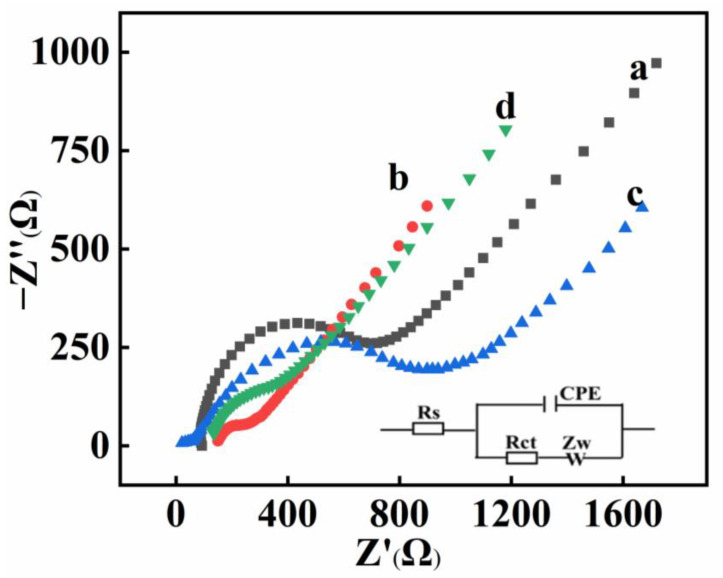
Electrochemical impedance spectroscopy of bare GCE (a), BC/GCE (b), MnMgFe-LDHs/GCE (c), and MnMgFe-LDHs/BC/GCE (d).

Electrochemical impedance spectroscopy (EIS) was employed to study the interface behavior of the modified electrode. Figure 2 demonstrates the Nyquist plots for different electrodes with a simulated equivalent circuit, where *R*_ct_, Cdl, Rs, and Zw represent charge transfer resistance, double layer capacitance, solution resistance, and Warburg impedance, respectively. The *R*_ct_ value of the fabricated electrode is estimated on the basis of the diameter of the semicircle in the high-frequency region. By calculation, the *R*_ct_ values of bare glass carbon electrode (GCE), BC/GCE, MnMgFe-LDHs/GCE, and MnMgFe-LDHs/BC/GCE are 623.7 Ω, 87.8 Ω, 913.2 Ω, and 204.6 Ω, respectively. Obviously, BC/GCE displays a lower Rct, while MnMgFe-LDHs/GCE shows a larger Rct in comparison with bare GCE, suggesting the good electronic conductivity of BC and the semiconductor properties of MnMgFe-LDHs. However, the combination of BC and MnMgFe-LDHs causes a lower Rct than that of MnMgFe-LDHs/GCE. The result demonstrates that the incorporation of BC into MnMgFe-LDHs significantly elevates the electron transfer efficiency.

**Figure 3:**
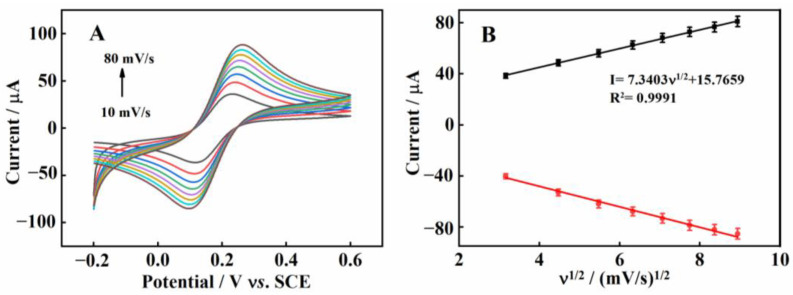
(**A**) CV curves of MnMgFe-LDHs/BC/GCE under different scan rates in 0.1 mol/L KCl containing 5 mmol/L [Fe(CN)_6_]^3−/4−^; (**B**) the plot of the peak current and *v*^1/2^.

Then, the effective surface area of MnMgFe-LDHs/BC/GCE was measured by cyclic voltammetry (CV) at different scan rates. Obviously, the peak current increases with increasing the scan rate from 10 to 80 mV/s (Figure 3A). The plot of the peak current versus the square root of the scan rate (*v*^1/2^) exhibits a good linear correlation with a regression equation of I = 7.340 *v* ^1/2^ + 15.766 (R^2^ = 0.999) (shown in Figure 3B). The effective surface area of MnMgFe-LDHs/BC/GCE can be calculated through the Randles–Sevcik equation (Equation (1)) [21]:

I_p_ = 2.69 × 10^5^ n^3/2^ A D_0_^1/2^ C_0_
*v*^1/2^ (1)

where I_p_, n, A, D_0_, and C_0_ represent anodic peak current (A), electron-transfer number, the effective surface area of electrode, diffusion coefficient of K_3_[Fe(CN)_6_] in 0.1 mol/L KCl (0.76 × 10^−5^ cm^2^ s^−1^), and the concentration of K_3_[Fe(CN)_6_] (mol cm^−3^), respectively. The effective surface area of MnMgFe-LDHs/BC is calculated to be 0.091 cm^2^. The large effective surface area could endow MnMgFe-LDHs/BC with abundant adsorption sites and excellent adsorption capacity for Cd^2+^ detection.

### 2.3. Electrochemical Behaviors of Different Electrodes

**Figure 4:**
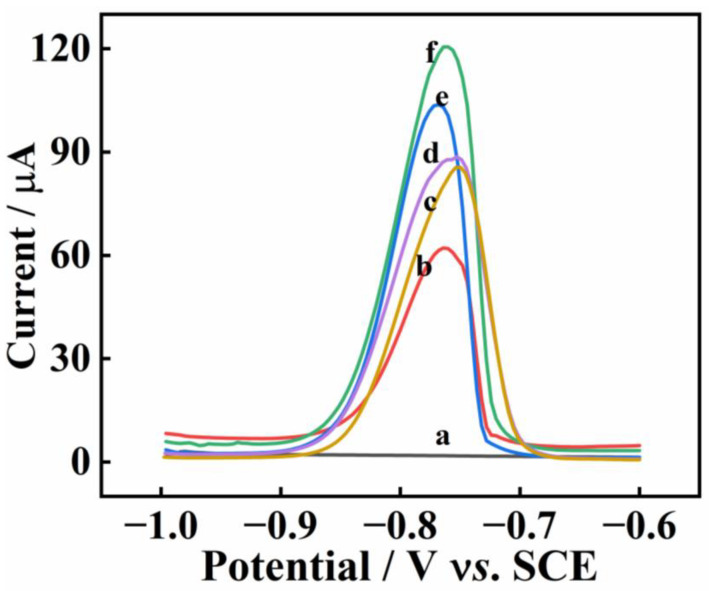
DPASV of 100 μg/L Cd^2+^ at bare GCE (a), BC/GCE (b), MnFe-LDHs/GCE (c), MgFe-LDHs/GCE (d), MnMgFe-LDHs/GCE (e), and MnMgFe-LDHs/BC/GCE (f) in ABS (pH 5.0).

The differential pulse anodic stripping voltammetry (DPASV) response signals of diverse electrodes were tested to examine their electroanalytical properties toward Cd^2+^. Figure 4 illustrates the electrochemical behaviors of bare GCE (a), BC/GCE (b), FeMn-LDHs/GCE (c), FeMg-LDHs/GCE (d), MnMgFe-LDHs/GCE (e), and MnMgFe-LDHs/BC/GCE (f) in ABS (pH 5.0) against 100 µg/L Cd^2+^. As shown from Figure 4, no visible stripping peak is observed at bare GCE, while BC/GCE shows an obvious stripping peak, which could be attributed to its outstanding conductivity and the large electrochemical effective area. At LDHs-modified GCEs, there are much larger peak currents due to their good adsorption properties for Cd^2+^. In particular, the peak current of MnMgFe-LDHs/GCE is significantly larger than that of MnFe-LDHs/GCE and MgFe-LDHs/GCE, mainly ascribing to its good electrical conductivity. Furthermore, the synergistic effect between BC and MnMgFe-LDHs causes the MnMgFe-LDHs/BC/GCE’s highest current, indicating that the developed MnMgFe-LDHs/BC/GCE can be used as the promising electrochemical sensing platform for Cd^2+^ detection.

### 2.4. Optimization of Experimental Parameters

**Figure 5:**
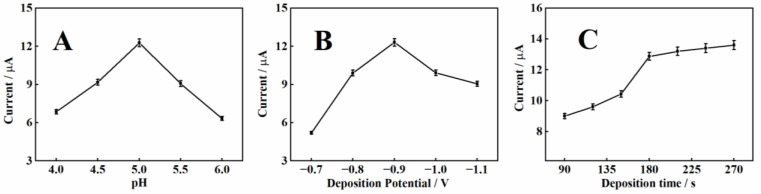
Effect of (**A**) pH value of ABS, (**B**) electrochemical deposition potential, and (**C**) deposition time on the peak current of Cd^2+^ at MnMgFe-LDHs/BC/GCE.

The experimental conditions (pH, deposition potential, and deposition time) at MnMgFe-LDHs/BC/GCE were studied to achieve the maximum peak currents for Cd^2+^ detection. The pH value of the buffer solution was optimized in Figure 5A. As shown, the stripping peak current increases with increasing pH from 4.0 to 5.0, which is attributed to the decrease in electrostatic repulsion as the H^+^ concentration decreases. The maximum peak current is found when pH is 5.0. The stripping peak current decreases sharply when the pH value is further increased, which is due to the hydrolysis of Cd^2+^. Therefore, 5.0 is used as the optimal pH of ABS.

The effect of deposition potential on the stripping peak current of Cd^2+^ was researched over the potential range from −0.7 to −1.1 V (Figure 5B). When the deposition potential shifts from −0.7 to −0.9 V, the electrochemical reduction for Cd^2+^ is promoted, and then the stripping peak current increases significantly. However, the peak current gradually decreases when the deposition potential is more negative than −0.9 V, which could be attributed to the fact that the hydrogen evolution has taken place [22]. Thus, −0.9 V is considered the optimal potential.

Similarly, the effect of deposition time was explored in the range from 90 to 270 s. As shown in Figure 5C, the peak current gradually enhances as the deposition time increases from 90 to 180 s, which could be due to the increased amount of Cd^2+^ deposited onto the electrode surface. However, the stripping current reaches a plateau as the deposition time goes beyond 180 s, owing to the saturation of active sites on MnMgFe-LDHs/BC/GCE. Thus, 180 s is selected as the optimal deposition time.

### 2.5. DPASV Detection of Cd^2+^ at MnMgFe-LDHs/BC/GCE

**Figure 6:**
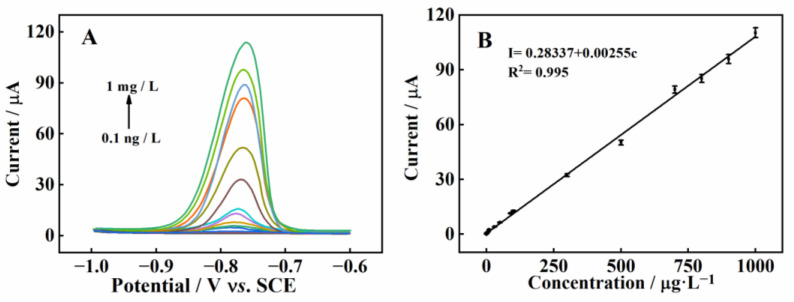
(**A**) DPASV responses of MnMgFe-LDHs/BC/GCE in ABS (pH 5.0) containing different concentrations of Cd^2+^. (**B**) Calibration curve between the stripping peak current and Cd^2+^ concentration.

The DPASV responses of MnMgFe-LDHs/BC/GCE toward different concentrations of Cd^2+^ were measured to evaluate the analytical capability of this constructed sensor under the optimized experimental parameters. From Figure 6A, the stripping peak signal of Cd^2+^ gradually increases with the increase in the Cd^2+^ concentration. The peak current (I) is linearly proportional to Cd^2+^ concentration from 0.1 ng/L−1.0 mg/L, with the linear regression equation of I = 0.283 + 0.003 c (R^2^ = 0.995) (Figure 6B). The limit of detection is calculated as 0.03 ng/L with a signal-to-noise ratio of 3, which is lower than those values reported at NH_2_-Ti_3_C_2_T_x_/screen-printed carbon electrode (0.48 μg L^−1^) [23], Fe_3_O_4_/Bi_2_O_3_/C_3_N_4_/GCE (337.2 ng L^−1^) [24], and graphdiyne/GCE (51.7 ng L^−1^) [25]. The good detection performance might be derived from the following collective effects: (i) the large specific surface area of MnMgFe-LDHs/BC would provide abundant attachment sites for the enrichment of Cd^2+^; (ii) the abundant surface-hydroxyl groups and various interlayer anions inside MnMgFe-LDHs enable a greater chance of trapping Cd^2+^; (iii) the in situ anchoring of MnMgFe-LDHs particles on BC with excellent conductivity would effectively improve the conductivity of MnMgFe-LDHs/BC, ultimately facilitating the electron transfer between Cd^2+^ and the electrode surface.

### 2.6. Repeatability, Reproducibility, and Selectivity of MnMgFe-LDHs/BC/GCE

**Figure 7:**
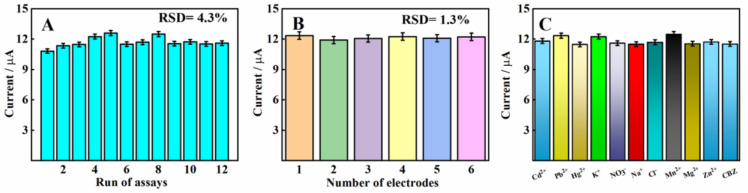
(**A**) Repeatability research of MnMgFe-LDHs/BC/GCE. (**B**) Reproducibility research on six independent MnMgFe-LDHs/BC/GCEs. (**C**) Selectivity research of MnMgFe-LDHs/BC/GCE.

The repeatability of MnMgFe-LDHs/BC/GCE was assessed via using one modified electrode for 12 consecutive measurements toward 100 μg/L Cd^2+^. As shown in Figure 7A, the current responses remain almost constant, with a relative standard deviation (RSD) of 4.3%, demonstrating that the repeatability of MnMgFe-LDHs/BC/GCE is satisfactory. Additionally, to research the reproducibility of MnMgFe-LDHs/BC/GCE (Figure 7B), six parallel MnMgFe-LDHs/BC/GCEs were used to detect 100 μg/L Cd^2+^ under the same conditions. The RSD of the current responses is calculated to be 1.3%, demonstrating that the reproducibility of the proposed sensor is good.

To explore the selectivity of the MnMgFe-LDHs/BC/GCE, the DPASV response of 100 μg/L Cd^2+^ was measured in the presence of interfering substances and without interfering substances. As depicted in Figure 7C, the effect of 50-fold excess concentration of Pb^2+^, Hg^2+^, K^+^, NO_3_^−^, Na^+^, Cl^−^, Mn^2+^, Mg^2+^, Zn^2+^, and CBZ on the response signal of Cd^2+^ is almost negligible. The results indicate that MnMgFe-LDHs/BC/GCE possesses excellent anti-interference ability.

### 2.7. Application of MnMgFe-LDHs/BC/GCE for Cd^2+^ Detection in Real Sample Analysis

The practical feasibility and reliability of the produced sensors were assessed by testing Cd^2+^ in a paddy water sample. The sample was collected from Jiangxi Agricultural University, then filtered with a 0.22 μm filter membrane to remove the impurities and diluted 100 times with 0.1 mol/L ABS (pH 5.0), which was used as the real sample. Subsequently, various concentrations of Cd^2+^ solution were added into the real sample and analyzed quantitatively by DPASV to research the recovery (Table 1). The result shows the recovery of Cd^2+^ in paddy water sample ranges from 97.3% to 102.3%, with the RSD less than 4.3%, which demonstrates that the accuracy of the proposed MnMgFe-LDHs/BC/GCE toward Cd^2+^ in water samples is satisfactory.

### 2.8. Adsorption Properties of MnMgFe-LDHs/BC toward Cd^2+^

**Figure 8:**
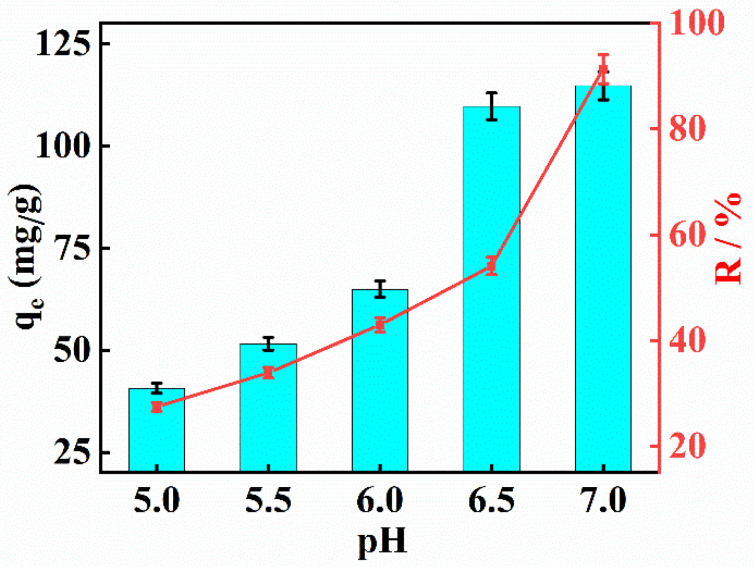
Relationship between solution pH and adsorption performance of Cd^2+^ by MnMgFe-LDHs/BC.

The MnMgFe-LDHs/BC composite was used to remove Cd^2+^ from the water. The effect of pH on Cd^2+^ removal by MnMgFe-LDHs/BC composite was firstly investigated (Figure 8). It shows that the adsorption capacity increases gradually at the beginning, and then reaches a plateau at the pH range of 5.0 to 7.0. Adsorption competition will happen between H^+^ and Cd^2+^ in the solution when the pH value is low, resulting in poor adsorption performance toward Cd^2+^. However, the negatively charged functional groups on the surface of MnMgFe-LDHs/BC increase with the increase in pH, which will provide a large number of functional adsorption sites for Cd^2+^. The high-level adsorption of Cd^2+^ on MnMgFe-LDHs/BC composite at pH 6.5–7.0 could be surface coprecipitation and/or a surface complexation reaction of Cd^2+^ with MnMgFe-LDHs/BC composite.

**Figure 9:**
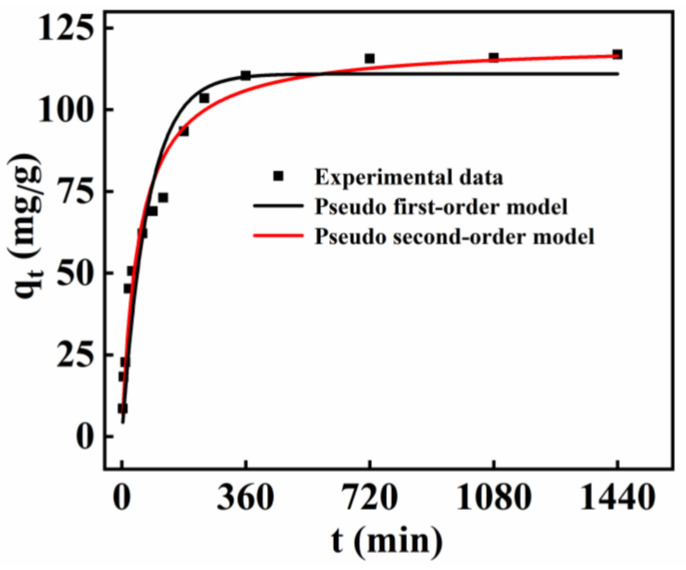
Influences of the uptake time and adsorption kinetics onto MnMgFe-LDH/BC of Cd^2+^.

The influence of adsorption time (0–24 h) on Cd^2+^ adsorption is shown in Figure 9. The adsorption capacity of Cd^2+^ on MnMgFe-LDH/BC displays a sharp rise within 5 h, followed with an attained equilibrium in about 6 h. The sharp rise on the first stage can be interpreted by the fact that the abundant binding sites on the surface of MnMgFe-LDH/BC were rapidly occupied by Cd^2+^. Whereas as time increased, the Cd^2+^ on MnMgFe-LDH/BC surface was saturated, so the adsorption capacity remained at the plateau.

All kinetic data are analyzed to fit the pseudo-first-order and pseudo-second-order models. The associated kinetic parameters determined from the two models are included in Table 2. The *R^2^* value (0.9750) for the pseudo-second-order kinetic model is much higher than that of pseudo-first-order (0.9354), demonstrating that the pseudo-second-order model had a superior fit compared to the pseudo-first-order model for the adsorption of Cd^2+^ on MnMgFe-LDH/BC. The applicability of the pseudo-second-order kinetic model shows that the chemical adsorption process is the key control step in the adsorption process of Cd^2+^ on MnMgFe-LDH/BC. The expression Equations (Equations (2) and (3)) of pseudo-first-order and pseudo-second-order models are given as follows:

(2) $$q_{t}=q_{e}\left(1-e^{-k_{1}t}\right)$$

(3) $$q_{t}=q_{e}^{2}k_{2}t/\left(1+q_{e}k_{2}t\right)$$

where *q_t_* (mg/g) and *q_e_* (mg/g) are the adsorption capacity at time (*t*) and the adsorption equilibrium, respectively. The *k_1_* (min^−1^) and *k_2_* (g/mg·min) are the pseudo-first- and pseudo-second-order rate constants, respectively.

**Figure 10:**
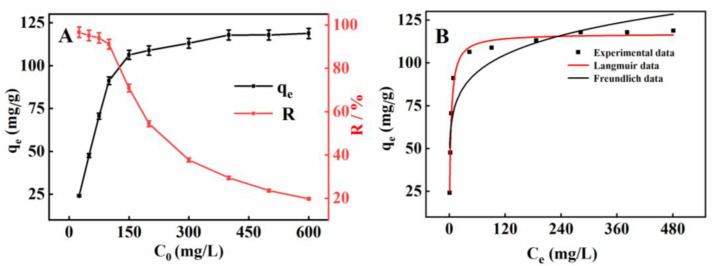
(**A**) Relationship between initial Cd^2+^ concentration and adsorption as well as removal efficiency; (**B**) adsorption isotherm study of Cd^2+^ removal over FeMnMg-LDH/BC composite.

The relationship between equilibrium adsorption capacity and initial Cd^2+^ concentrations is presented in Figure 10A. With the initial concentration of Cd^2+^ increasing from 0 to 600 mg/L, the equilibrium adsorption capacity of MnMgFe-LDHs/BC on Cd^2+^ increases rapidly at first and then levels off, which is due to the gradual saturation or even depletion of binding sites. On the other hand, it is observed that the removal efficiency decreases steadily. The availability of sufficient adsorption sites contributes to the effective removal of Cd^2+^ at lower concentrations. When C_0_ is higher than 75 mg/L, the removal efficiency decreases, which could be attributed to the fact that almost all the adsorption sites on the surface of MnMgFe-LDHs/BC have been occupied. Considering both the removal efficiency and adsorption capacity, 75 mg/L is thought to be the optimal initial Cd^2+^ concentration for MnMgFe-LDHs/BC.

Langmuir (Equation (4)) and Freundlich (Equation (5)) adsorption isotherm models are employed to study the adsorption type of MnMgFe-LDHs/BC toward Cd^2+^, which are listed according to [26,27]:

(4) $$q_{e}=q_{m}C_{e}K_{L}/\left(1+C_{e}K_{L}\right)$$

(5) $$q_{e}=K_{f}C_{e}{}^{1/n}$$

where *q_e_* (mg/g) is the equilibrium adsorption amount of Cd^2+^. Ce is the equilibrium concentration (mg/L). *q_max_* (mg/g) represents the theoretical maximum adsorption capacity of MnMgFe-LDHs/BC toward Cd^2+^. *K_L_* (L/mg) is the Langmuir constant correlated with intensity of adsorption. *K_f_* (mg^(1−n)^/L^−n^/g) and n are the Freundlich constant matching with capacity and intensity of adsorption.

Next, the equilibrium adsorption isotherm of MnMgFe-LDHs/BC toward Cd^2+^ was studied and the result shows that it matches better with the Langmuir model (R^2^ = 0.9919) than the Freundlich model (R^2^ = 0.8294) (Figure 10B), demonstrating that the adsorption of MnMgFe-LDHs/BC toward Cd^2+^ belongs to a uniform monolayer adsorption process. Based on this, the theoretical *q_max_* is calculated to be 117 mg/g by the Langmuir model, which is consistent with the experimental values. Compared with other adsorbents, MnMgFe-LDHs/BC shows better adsorption performance (Table 3), revealing that MnMgFe-LDHs/BC has a great potential to be a low-cost and high-efficiency adsorbent.

**Figure 11:**
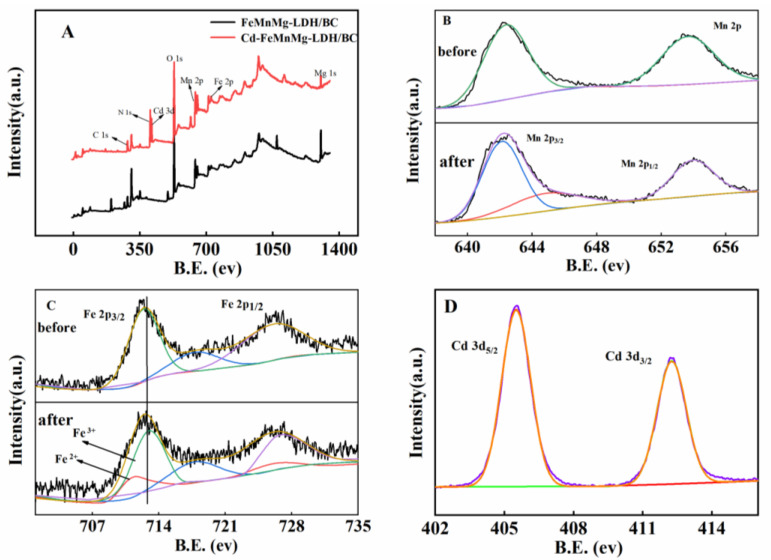
(**A**) Full range of XPS spectra of MnMgFe-LDHs/BC, (**B**) Mn 2p spectrums of MnMgFe-LDHs/BC, (**C**) Fe 2p of MnMgFe-LDHs/BC before and after adsorption with Cd^2+^, (**D**) Cd 3d spectrums of MnMgFe-LDHs/BC.

XPS analysis of MnMgFe-LDHs/BC before and after adsorption was performed to further investigate the adsorption mechanism toward Cd^2+^. As shown in Figure 11A, the XPS wide-scan survey of MnMgFe-LDHs/BC reveals that there are C, N, O, Mn, Fe, and Mg elements. However, there is a new peak on the full spectra of MnMgFe-LDHs/BC after adsorbing Cd^2+^, which is derived from Cd element. For the high-resolution spectrum of Mn 2p, it shows that there are Mn^2+^ and Mn^3+^ in MnMgFe-LDHs/BC. However, there is a satellite peak after the adsorption of Cd^2+^ (Figure 11B), which is offset from the main peaks of the Mn 2p doublet by approximately 2.5 eV toward higher binding energies [27]. It demonstrates that Mn^2+/3+^ is oxidated to Mn^3+/4+^ during the adsorption process. Meanwhile, the high-resolution spectrum of Fe 2p shows there are two peaks at 711.8 and 725.3 eV (Figure 11C), which are consistent with the Fe^3+^ of Fe(OH)O [35]. Moreover, the satellite peaks at 719.2 and 732.7 eV further reveal the exist of Fe^3+^. Subsequently, a new peak of Fe^2+^ appears at 710.7 eV after the adsorption of Cd^2+^. The above results show that there is a self-oxidation-reduction reaction on the surface of MnMgFe-LDHs/BC during the adsorption process of Cd^2+^. The high-resolution spectrum of O 1s shows that the peaks at 533.2 and 533.6 eV are shifted after the adsorption of Cd^2+^, which might be caused by the change of the O atom environment due to the formation of a complex between the oxygen-containing functional group and Cd^2+^. As for the high-resolution spectrum of Cd 3d (Figure 11D), there are two peaks, at 405.1 and 411.8 eV, which match well with the characteristic peak of Cd(OH)_2_, evidencing that there is an induced precipitation of Cd^2+^ on the surface of MnMgFe-LDHs/BC. On the basis of all the above analyses, it can be concluded that the effective removal of Cd^2+^ by MnMgFe-LDHs/BC is due to the synergistic effect of surface adsorption, surface-induced precipitation, and complexation [17].

### 2.9. Regeneration and Stability

**Figure 12:**
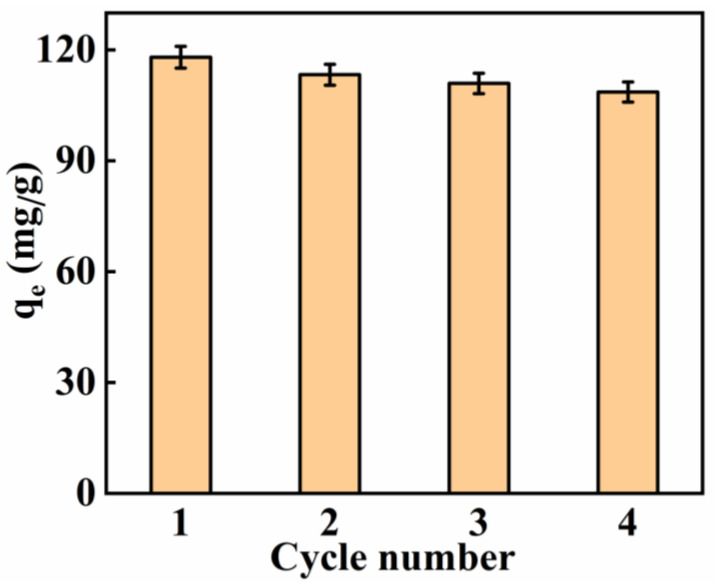
Adsorption capacity of regenerated adsorbent for four cycles.

The regeneration of the prepared MnMgFe-LDHs/BC was studied. Figure 12 depicts the adsorption capacity of MnMgFe-LDHs/BC adsorbent for Cd^2+^ in function of the adsorption–desorption cycles. As can be seen, the adsorbent remains above 90% of its initial adsorption capacity after four recycles, demonstrating the reliable recyclability and good stability for removal of Cd^2+^ from water sample.

The TCLP experiment was also applied to study the stability of MnMgFe-LDHs/BC and the treatment process. The procedure for the TCLP experiment is described in Section 3.7. Result shows that the dissolved Cd^2+^ accounted for 0.4% of the Cd^2+^ in MnMgFe-LDHs/BC@Cd^2+^, demonstrating that MnMgFe-LDHs/BC owns strong immobilization ability to Cd^2+^ and would not cause secondary pollution. It further proves that MnMgFe-LDHs/BC is an outstanding adsorption material for Cd^2+^.

## 3. Experimental Section

### 3.1. Materials

Rape stalks were harvested from the experimental field in Jiangxi Agricultural University (Nanchang, China). Analytical grade Mn(NO_3_)_2_, Mg(NO_3_)_2_, Fe(NO_3_)_3_, Cd(NO_3_)_2_, NaOH, HCl, and HNO_3_ were obtained from Huakun Chemical Reagents Co., Ltd. (Nanchang, China). Furthermore, 0.1 mol/L acetate buffer solution was prepared by sodium acetate and acetic acid. All reagents in this work were used without further purification.

### 3.2. Apparatus

Scanning electron microscopy (SEM/Zeiss Gemini 300, Germany), X-ray diffraction (XRD/D8 Advance, Germany), and X-ray photoelectron spectroscopy (XPS/Thermo Fischer Escalab 250Xi, Thermo Fisher Scientific Inc., Waltham, USA) were used for the characterization of morphology and structure. All electrochemical experiments were carried out with a CHI 760E electrochemical workstation (CH Instrument Co., Ltd., Shanghai, China) with a three-electrode system consisting of a working electrode (GCE with diameter of 3 mm), a counter electrode (platinum wire), and a reference electrode (saturated calomel electrode (SCE)).

### 3.3. Preparation of BC and LDHs/BC Composite

Rape stalks-derived BC was produced based on the previous method [7]. The rape stalks were chopped, washed, dried, and then activated with KOH solution. After that, the activated rape stalks were pyrolyzed at 800 °C for 2 h under N_2_ with a heating rate of 5 °C/min. The resulting BC was ground into powder.

MnMgFe-LDHs/BC was synthesized according to the following steps. First, 0.02 mol Mn(NO_3_)_2_, 0.03 mol Mg(NO_3_)_2_, 0.01 mol Fe(NO_3_)_3_, and 1 g BC were added into 50 mL ultrapure water to obtain a mixed solution and stirred for 1 h. Next, 0.5 mol/L NaOH was dropped into the above solution under stirring until the pH of the solution was 12. Then, the mixed solution was stirred vigorously for 2 h and aged under 60 °C for 24 h. Finally, the obtained precipitate was washed with ultrapure water 3 times and dried at 60 °C for 12 h. In addition, the MnMgFe-LDHs, MnFe-LDHs, and MgFe-LDHs were prepared using the same method without adding BC.

### 3.4. Preparation of the Modified Electrodes

First of all, GCE surface was polished with 0.3 μm alumina powder, and then washed with deionized water and ethanol through ultrasound. Subsequently, 5 μL MnMgFe-LDHs/BC dispersion (1 mg/mL) was dropped on bare GCE and dried at room temperature, represented as MnMgFe-LDHs/BC/GCE. For comparison, BC/GCE, MnFe-LDHs/GCE, and MgFe-LDHs/GCE were prepared with the same procedures.

### 3.5. Analytical Procedure

**Scheme 1:**
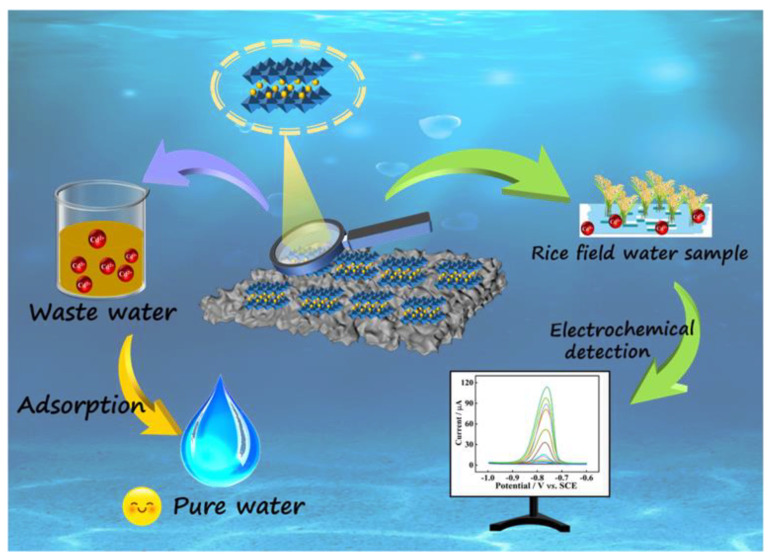
Schematic illustration of the use of MnMgFe-LDHs/BC for electrochemical detection and removal of Cd^2+^ in aqueous solution.

Differential pulse anodic stripping voltammetry (DPASV) was utilized to detect Cd^2+^. Briefly, the modified electrode was immersed in 0.1 mol/L NaAc-HAc buffer (pH, 5.0) containing a certain concentration of Cd^2+^, which was deposited at −0.9 V for 180 s with magnetic stirring. The DPASV method for Cd^2+^ detection is shown in Scheme 1.

### 3.6. Adsorption Studies of Cd^2+^

The adsorption experiment was carried out by immersing 250 mg MnMgFe-LDHs/BC in 25 mL Cd^2+^ solution at various concentrations (0 to 600 mg/L). Here, the pH of the Cd^2+^ solution was adjusted with HNO_3_ or NaOH. The mixture was then stirred for a specific time under room temperature. After that, the suspension was filtered through a syringe filter, and the Cd^2+^ concentration in the filtrate was detected by atomic absorption spectrophotometer (ASS). The adsorbed amount (q, mg/g) and removal efficiency (R, %) of Cd^2+^ were calculated according to Equations (6) and (7), respectively.

(6) $$q=\frac{\left(C_{0}-C_{t}\right)V}{m}$$

(7) $$R=\frac{\left(C_{0}-C_{t}\right)}{C_{0}}\times 100\%$$

where q (mg/g) represents the mass of the Cd^2+^ adsorbed by each gram of MnMgFe-LDHs/BC. *C*_0_ (mg/L) and *C*_e_ (mg/L) denote the initial Cd^2+^ concentration and the equilibrium concentrations of Cd^2+^, respectively. *V* (L) denotes the Cd^2+^ volume. *m* (g) is the dose of MnMgFe-LDHs/BC.

### 3.7. TCLP Experiment

First of all, 5.7 mL glacial acetic acid was diluted to 1 L with deionized water, and the pH value was adjusted to 2.88 to obtain TCLP extract solution. Then, 2 g of MnMgFe-LDHs/BC@Cd^2+^ was mixed with 40 mL TCLP extract, and the dispersion was oscillated for 18 h at room temperature. After shaking, the dispersion was centrifuged at 4000 r/min for 20 min and filtered with 0.45 μm filter membrane to collect supernatant. Finally, the content of Cd^2+^ in the supernatant was determined by ASS.

## 4. Conclusions

In summary, a new MnMgFe-LDHs/BC composite was prepared through a coprecipitation method. The resulted MnMgFe-LDHs/BC presents large specific surface area, porous channels, and fast electron transfer, enabling high enrichment capacity for Cd^2+^ and the improved electrochemical “deposition-stripping” process. Under the synergistic effect of MnMgFe-LDHs/BC and BC, a wide linear range from 0.1 ng/L to 1.0 mg/L and a low detection limit of 0.03 ng/L are achieved on the MnMgFe-LDHs/BC-based sensor. Furthermore, satisfactory repeatability, reproducibility, and selectivity are obtained, and the as-prepared sensor is suitable for detecting Cd^2+^ in paddy water. Furthermore, as the adsorbent, MnMgFe-LDHs/BC composite displays favorable removal performance for Cd^2+^ in aqueous solution. All these results indicate MnMgFe-LDHs/BC composite could be used as a promising platform for electrochemical detection and removal of Cd^2+^ in water environments.

## Figures and Tables

**Table 1:** Recovery research of Cd^2+^ in paddy water samples by MnMgFe-LDHs/BC/GCE (n = 3).

Sample	Added (μg/L)	Found (μg/L)	Recovery (%)	RSD (%)
1	0	-	-	-
2	0.05	0.05 ± 0.002	97.3	4.3
3	0.50	0.50 ± 0.021	100.6	4.1
4	5.00	5.00 ± 0.140	99.6	2.9
5	50.00	50.00 ± 1.320	102.3	1.8

**Table 2:** Adsorption kinetics parameters for adsorption of Cd^2+^ on MnMgFe-LDH/BC.

	Pseudo-First-Order Model			Pseudo-Second-Order Model		
*q_e,exp_* (mg/g)	*k_1_* (min^−1^)	R^2^	*q_e_* (mg/g)	*k_2_* (g/mg·min)	R^2^	*q_e_* (mg/g)
118.00	0.0133	0.9354	110.97	0.000168	0.9750	117.94

**Table 3:** Comparison of Cd^2+^ adsorption capacities with some other adsorbents.

Adsorbents	Adsorption Capacity (mg/g)	Ref.
Kiwi branch BC/MgFe-LDH	25.60	[28]
Peanut husk BC	28.99	[29]
Mg-Al-CO_3_-LDH	70.20	[30]
MgAL-LDH@graphene oxide-SH	102.77	[31]
Mango kernel BC	27.81	[32]
Garden waste BC	40.00	[33]
BC-Fe-S	57.71	[34]
MnMgFe-LDH/BC	118.00	This work

## Data Availability

The data presented in this study are available in the article.

## Sample Availability

Samples of the compounds are not available from the authors.
